# Crystal structure of *catena*-poly[[[di­aqua­cobalt(II)]-bis­(μ-hex-3-enedi­nitrile-κ^2^ *N*:*N*′)] bis­(tetra­fluorido­borate)]

**DOI:** 10.1107/S2056989015009548

**Published:** 2015-05-23

**Authors:** Jung-Su Son, Sung-Chul Lim, Hochun Lee, Seung-Tae Hong

**Affiliations:** a Daegu Gyeongbuk Institue of Science & Technology (DGIST), Daegu 711-873, Republic of Korea

**Keywords:** crystal structure, cobalt, hex-3-enedi­nitrile, hydrogen bonding

## Abstract

In the structure of the title salt, [Co(C_6_H_6_N_2_)_2_(H_2_O)_2_](BF_4_)_2_, the Co^II^ atom is located on an inversion centre. The transition metal is in a slightly distorted octa­hedral coordination environment, defined by the cyano N atoms of four hex-3-enedi­nitrile ligands in equatorial positions and the O atoms of two water mol­ecules in axial positions. The bridging mode of the hex-3-enedi­nitrile ligands leads to the formation of cationic chains extending parallel to [1-10]. The BF_4_^−^ counter-anion is disordered over two sets of sites [occupancy ratio = 0.512 (19):0.489 (19)]. It is located in the voids between the cationic chains and is connected to the aqua ligands of the latter through O—H⋯F hydrogen bonds. One methyl­ene H atom of the hex-3-enedi­nitrile ligand forms another and weak C—H⋯O hydrogen bond with a water O atom of a neighbouring chain, thus consolidating the three-dimensional network structure.

## Related literature   

Aliphatic di­nitriles have gained much attention not only due to their rich coordination chemistry with transition-metal ions (Storhoff & Lewis, 1977 ▸; Heller & Sheldrick, 2004 ▸; Blount *et al.*, 1969 ▸), but also due to their applications as functional electrolyte additives for lithium ion batteries (Kim *et al.*, 2011 ▸, 2014*a* ▸,*b* ▸). While the coordination complexes of saturated aliphatic di­nitrile ligands have been extensively studied (Storhoff & Lewis, 1977 ▸; Heller & Sheldrick, 2004 ▸; Blount *et al.*, 1969 ▸), those of unsaturated di­nitrile ligands like in the title compound have hardly been reported so far. 
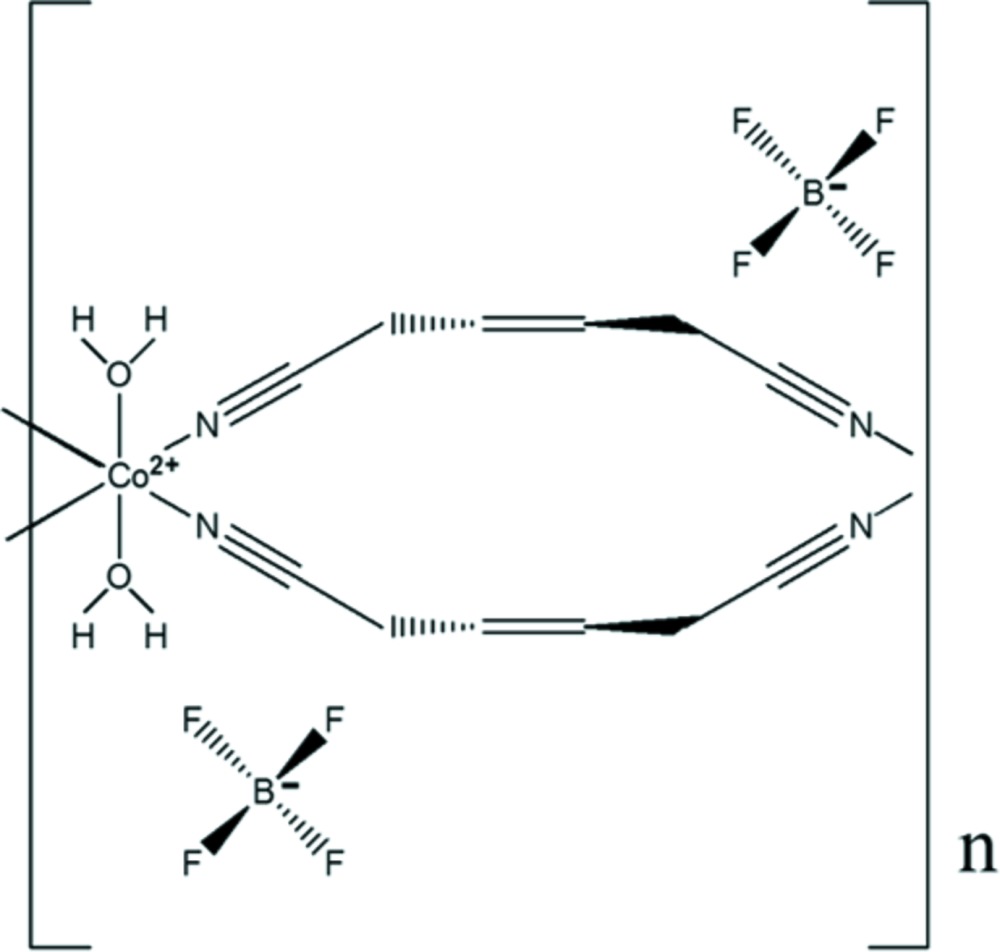


## Experimental   

### Crystal data  

[Co(C_6_H_6_N_2_)(H_2_O)_2_](BF_4_)_2_
*M* *_r_* = 480.84
Triclinic, 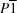

*a* = 7.9839 (11) Å
*b* = 8.3434 (11) Å
*c* = 8.8441 (13) Å
α = 71.380 (5)°
β = 88.458 (5)°
γ = 66.184 (4)°
*V* = 507.21 (12) Å^3^
*Z* = 1
Mo *K*α radiation
μ = 0.93 mm^−1^
*T* = 103 K
0.20 × 0.20 × 0.20 mm

### Data collection  

Bruker APEXII CCD diffractometer
Absorption correction: multi-scan (*SADABS*; Bruker, 2006 ▸) *T*_min_ = 0.60, *T*_max_ = 0.75
14705 measured reflections
2501 independent reflections
2233 reflections with *I* > 2σ(*I*)
*R*_int_ = 0.038

### Refinement  

*R*[*F* ^2^ > 3σ(*F* ^2^)] = 0.033
*wR*(*F* ^2^) = 0.068
*S* = 0.87
2202 reflections
170 parameters
20 restraints
H-atom parameters constrained
Δρ_max_ = 0.83 e Å^−3^
Δρ_min_ = −0.62 e Å^−3^

### 

Data collection: *APEX2* (Bruker, 2006 ▸); cell refinement: *SAINT* (Bruker, 2006 ▸); data reduction: *SAINT*; program(s) used to solve structure: *SHELXS97* (Sheldrick, 2008 ▸); program(s) used to refine structure: *CRYSTALS* (Betteridge *et al.*, 2003 ▸); molecular graphics: *ATOMS* (Dowty, 2000 ▸); software used to prepare material for publication: *CRYSTALS*.

## Supplementary Material

Crystal structure: contains datablock(s) I. DOI: 10.1107/S2056989015009548/wm5160sup1.cif

Structure factors: contains datablock(s) I. DOI: 10.1107/S2056989015009548/wm5160Isup2.hkl

**Fig. 1.**
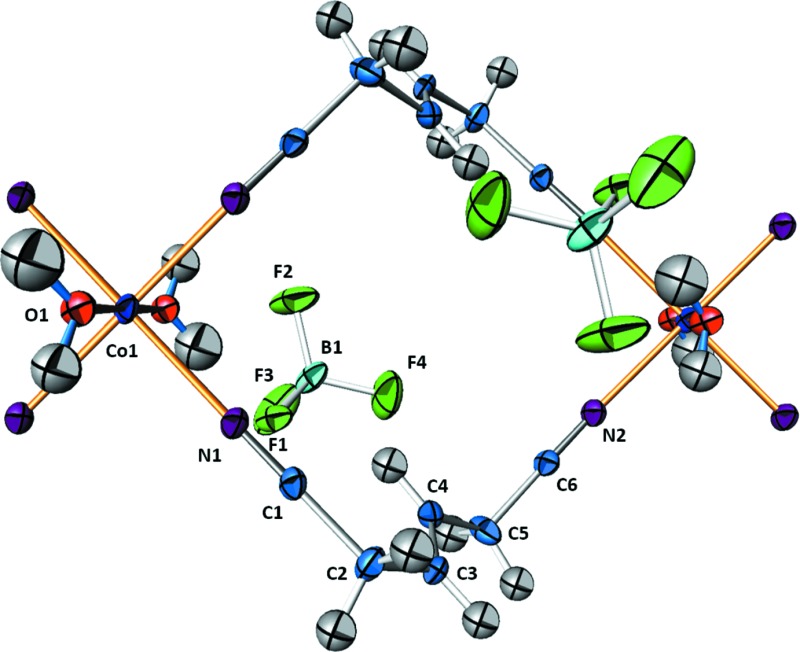
The cationic chain structure of the title compound with displacement ellipsoids drawn at the 50% probability level. The BF4- anion is shown only with the major part of the disorder.

4 − . DOI: 10.1107/S2056989015009548/wm5160fig1.tif
The cationic chain structure of the title compound with displacement ellipsoids drawn at the 50% probability level. The BF_4_^−^ anion is shown only with the major part of the disorder.

**Fig. 2.**
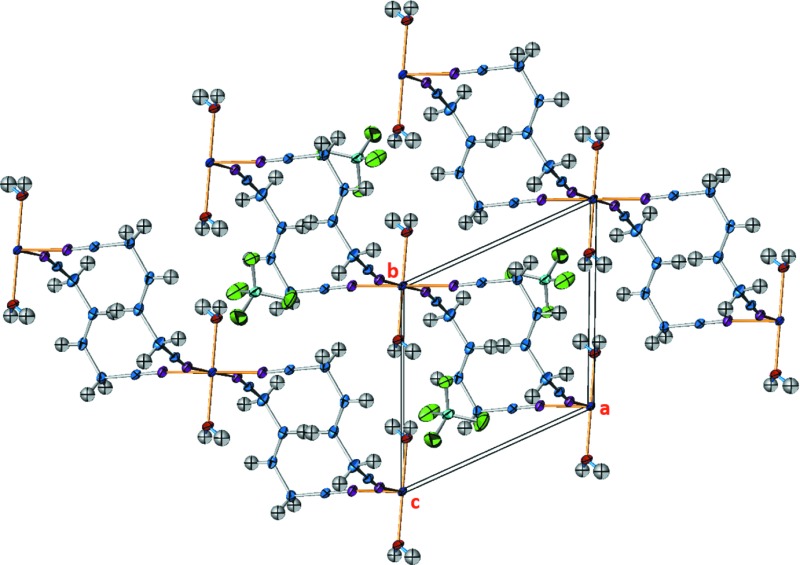
The crystal packing of the title compound with displacement ellipsoids drawn at the 50% probability level. The BF4- anion is shown only with the major part of the disorder. (Colour code: dark blue: Co, purple: N, blue: C, red: O, cyan: B, green: F, grey: H).

4 − . DOI: 10.1107/S2056989015009548/wm5160fig2.tif
The crystal packing of the title compound with displacement ellipsoids drawn at the 50% probability level. The BF_4_^−^ anion is shown only with the major part of the disorder. (Colour code: dark blue: Co, purple: N, blue: C, red: O, cyan: B, green: F, grey: H).

CCDC reference: 1401602

Additional supporting information:  crystallographic information; 3D view; checkCIF report

## Figures and Tables

**Table 1:** Hydrogen-bond geometry (, )

*D*H*A*	*D*H	H*A*	*D* *A*	*D*H*A*
C2H22O1^i^	0.97	2.52	3.348(3)	143(1)
O1H12F1^ii^	0.83	1.89	2.72(2)	175(1)
O1H11F2^i^	0.82	1.87	2.669(13)	163(1)

Symmetry codes: (i) 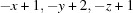; (ii) 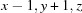.

## supplementary crystallographic information

### S1. Experimental

A solvent was prepared first by mixing ethyl­ene carbonate (EC) and ethyl methyl carbonate (EMC) in an 1:2 volume ratio (3.3 ml and 6.6 ml, respectively). 0.934 g of lithium tetra­fluoridoborate (LiBF_4_) were added to the solvent, and it was stirred for about 5 hours to dissolve the salt completely to form an 1M solution. 0.60 g (5 wt.%) of cobalt(II) tetra­fluoridoborate hexahydrate and 0.24 g (2 wt.%) hex-3-enedi­nitrile were added and dissolved in the solution. The solution was kept for 48 hours in an argon-atmosphere glove-box at room temperature, resulting in the growth of red crystals. The crystals were washed with pure EMC solvent three times in the argon-atmosphere glove-box.

### S2. Refinement

H atoms attached to C atoms of the title compound were placed in geometrically idealized positions and treated as rigid bodies with C—H distances constrained to 0.92–0.97 Å. Water H atoms were located from a difference map and refined with a distance of 0.82 Å. The BF_4_^-^ counter anion was refined with a positional disorder model where F2, F3 and F4 atoms are split into two positions while B1 and F1 atoms are not. Such a disorder model resulted in a slightly better refinement, reducing the *R*1 factor values from 0.041 to 0.033.

### Crystal data

**Table:**

[Co(C_6_H_6_N_2_)(H_2_O)_2_](BF_4_)_2_	*Z* = 1
*M**_r_* = 480.84	*F*(000) = 241
Triclinic, *P*1	*D*_x_ = 1.575 Mg m^−^^3^
Hall symbol: -P 1	Mo *K*α radiation, λ = 0.71073 Å
*a* = 7.9839 (11) Å	Cell parameters from 0 reflections
*b* = 8.3434 (11) Å	θ = 0–0°
*c* = 8.8441 (13) Å	µ = 0.93 mm^−^^1^
α = 71.380 (5)°	*T* = 103 K
β = 88.458 (5)°	Cuboid, yellow
γ = 66.184 (4)°	0.20 × 0.20 × 0.20 mm
*V* = 507.21 (12) Å^3^	

### Data collection

**Table:**

Bruker APEXII CCD diffractometer	2233 reflections with *I* > 2σ(*I*)
Graphite monochromator	*R*_int_ = 0.038
φ & ω scans	θ_max_ = 28.5°, θ_min_ = 2.8°
Absorption correction: multi-scan (*SADABS*; Bruker, 2006)	*h* = −10→10
*T*_min_ = 0.60, *T*_max_ = 0.75	*k* = −11→11
14705 measured reflections	*l* = −11→11
2501 independent reflections	

### Refinement

**Table:**

Refinement on *F*^2^	Primary atom site location: structure-invariant direct methods
Least-squares matrix: full	Hydrogen site location: difference Fourier map
*R*[*F*^2^ > 2σ(*F*^2^)] = 0.033	H-atom parameters constrained
*wR*(*F*^2^) = 0.068	Weighting scheme based on measured s.u.'s W = 1
*S* = 0.87	(Δ/σ)_max_ = 0.0002
2202 reflections	Δρ_max_ = 0.83 e Å^−^^3^
170 parameters	Δρ_min_ = −0.62 e Å^−^^3^
20 restraints	

### Special details

**Table:**

Geometry. All e.s.d.'s (except the e.s.d. in the dihedral angle between two l.s. planes) are estimated using the full covariance matrix. The cell e.s.d.'s are taken into account individually in the estimation of e.s.d.'s in distances, angles, and torsion angles; correlations between e.s.d.'s in cell parameters are only used when they are defined by crystal symmetry. An approximate (isotropic) treatment of cell e.s.d.'s is used for estimating e.s.d.'s involving l.s. planes.
Refinement. Refinement of *F*^2^ against ALL reflections. The weighted *R*-factor *wR* and goodness of fit *S* are based on *F*^2^, conventional *R*-factors *R* are based on *F*, with *F* set to zero for negative *F*^2^. The threshold expression of *F*^2^ > σ(*F*^2^) is used only for calculating *R*-factors(gt) *etc*. and is not relevant to the choice of reflections for refinement. *R*-factors based on *F*^2^ are statistically about twice as large as those based on *F*, and *R*- factors based on ALL data will be even larger.

### Fractional atomic coordinates and isotropic or equivalent isotropic displacement parameters (Å^2^)

**Table:**

	*x*	*y*	*z*	*U*_iso_*/*U*_eq_	Occ. (<1)
Co1	0.0000	1.0000	0.5000	0.0158	
N1	0.2687 (2)	0.8866 (2)	0.6299 (2)	0.0199	
N2	0.1190 (2)	0.9254 (2)	0.3036 (2)	0.0192	
C1	0.4140 (2)	0.8397 (3)	0.6904 (2)	0.0179	
C2	0.6004 (3)	0.7780 (3)	0.7691 (3)	0.0221	
C3	0.7036 (2)	0.5683 (3)	0.8382 (2)	0.0200	
C4	0.6448 (3)	0.4468 (3)	0.8245 (2)	0.0201	
C5	0.7535 (3)	0.2388 (3)	0.9045 (2)	0.0244	
C6	0.8244 (2)	0.1444 (2)	0.7878 (2)	0.0181	
O1	0.02105 (18)	1.25018 (18)	0.41358 (16)	0.0197	
B1	0.7569 (3)	0.7200 (3)	0.2131 (3)	0.0287	
F1	0.77132 (18)	0.55820 (17)	0.18696 (15)	0.0295	
F2	0.8571 (16)	0.6724 (15)	0.3523 (13)	0.0555	0.512 (19)
F3	0.8368 (13)	0.8076 (13)	0.0834 (11)	0.0630	0.512 (19)
F4	0.5804 (9)	0.8300 (10)	0.2014 (15)	0.0585	0.512 (19)
F21	0.9052 (8)	0.6606 (13)	0.3357 (11)	0.0255	0.489 (19)
F31	0.7671 (17)	0.8497 (11)	0.0853 (10)	0.0551	0.489 (19)
F41	0.5866 (9)	0.7929 (11)	0.2810 (16)	0.0534	0.489 (19)
H11	0.0413	1.2954	0.4771	0.034 (4)*	
H12	−0.0554	1.3393	0.3416	0.043 (4)*	
H21	0.5897	0.8291	0.8529	0.034 (4)*	
H22	0.6694	0.8293	0.6919	0.033 (4)*	
H31	0.8174	0.5253	0.8931	0.034 (4)*	
H41	0.5301	0.4889	0.7639	0.035 (4)*	
H51	0.6801	0.1848	0.9680	0.036 (4)*	
H52	0.8570	0.2189	0.9701	0.034 (4)*	

### Atomic displacement parameters (Å^2^)

**Table:**

	*U*^11^	*U*^22^	*U*^33^	*U*^12^	*U*^13^	*U*^23^
Co1	0.01363 (18)	0.01074 (17)	0.0198 (2)	−0.00181 (13)	−0.00123 (13)	−0.00526 (14)
N1	0.0166 (8)	0.0169 (8)	0.0222 (8)	−0.0025 (6)	−0.0014 (6)	−0.0070 (6)
N2	0.0170 (7)	0.0163 (8)	0.0211 (8)	−0.0039 (6)	0.0006 (6)	−0.0060 (6)
C1	0.0173 (7)	0.0138 (8)	0.0201 (9)	−0.0033 (7)	0.0015 (7)	−0.0067 (7)
C2	0.0153 (7)	0.0209 (9)	0.0292 (10)	−0.0055 (7)	−0.0023 (7)	−0.0093 (8)
C3	0.0139 (7)	0.0221 (9)	0.0194 (9)	−0.0018 (7)	−0.0025 (7)	−0.0081 (7)
C4	0.0170 (7)	0.0207 (9)	0.0178 (9)	−0.0024 (7)	0.0018 (7)	−0.0076 (7)
C5	0.0266 (7)	0.0216 (10)	0.0182 (9)	−0.0031 (8)	0.0026 (8)	−0.0074 (8)
C6	0.0172 (7)	0.0135 (8)	0.0176 (9)	−0.0032 (7)	−0.0023 (7)	−0.0015 (7)
O1	0.0223 (7)	0.0126 (6)	0.0219 (7)	−0.0052 (5)	−0.0021 (5)	−0.0056 (5)
B1	0.0252 (12)	0.0159 (10)	0.0380 (14)	−0.0057 (9)	−0.0119 (10)	−0.0028 (10)
F1	0.0363 (7)	0.0211 (6)	0.0283 (7)	−0.0096 (5)	−0.0101 (5)	−0.0067 (5)
F2	0.072 (4)	0.036 (3)	0.049 (4)	−0.010 (4)	−0.032 (4)	−0.017 (2)
F3	0.046 (3)	0.047 (4)	0.071 (3)	−0.026 (3)	−0.009 (3)	0.022 (3)
F4	0.0361 (18)	0.039 (3)	0.088 (5)	0.0039 (18)	0.000 (3)	−0.031 (3)
F21	0.0208 (19)	0.021 (2)	0.032 (2)	−0.0030 (16)	−0.0089 (16)	−0.0115 (15)
F31	0.072 (4)	0.031 (3)	0.046 (2)	−0.028 (3)	−0.025 (3)	0.019 (2)
F41	0.0304 (19)	0.042 (3)	0.088 (5)	−0.0067 (18)	0.010 (3)	−0.034 (3)

### Geometric parameters (Å, º)

**Table:**

Co1—N1^i^	2.1486 (17)	C4—H41	0.946
Co1—N2^i^	2.1050 (17)	C5—C6	1.460 (6)
Co1—O1^i^	2.0560 (15)	C5—H51	0.946
Co1—N1	2.1486 (17)	C5—H52	0.949
Co1—N2	2.1050 (17)	O1—H11	0.821
Co1—O1	2.0560 (15)	O1—H12	0.826
N1—C1	1.148 (6)	B1—F1	1.401 (3)
N2—C6^ii^	1.125 (5)	B1—F2	1.341 (12)
C1—C2	1.474 (8)	B1—F3	1.435 (11)
C2—C3	1.514 (9)	B1—F4	1.320 (9)
C2—H21	0.953	B1—F1	1.401 (3)
C2—H22	0.967	B1—F21	1.440 (12)
C3—C4	1.315 (4)	B1—F31	1.319 (11)
C3—H31	0.916	B1—F41	1.450 (10)
C4—C5	1.516 (3)		
			
N1^i^—Co1—N2^i^	90.57 (6)	C4—C3—H31	118.7
N1^i^—Co1—O1^i^	87.29 (6)	C3—C4—C5	122.3 (3)
N2^i^—Co1—O1^i^	91.15 (6)	C3—C4—H41	119.7
N1^i^—Co1—N1	179.995	C5—C4—H41	118.0
N2^i^—Co1—N1	89.4 (8)	C4—C5—C6	112.18 (14)
O1^i^—Co1—N1	92.7 (4)	C4—C5—H51	110.9
N1^i^—Co1—N2	89.4 (4)	C6—C5—H51	108.7
N2^i^—Co1—N2	179.995	C4—C5—H52	108.2
O1^i^—Co1—N2	88.9 (6)	C6—C5—H52	107.0
N1—Co1—N2	90.57 (6)	H51—C5—H52	109.8
N1^i^—Co1—O1	92.7 (8)	C5—C6—N2^ii^	178.3 (2)
N2^i^—Co1—O1	88.9 (3)	Co1—O1—H11	119.4
O1^i^—Co1—O1	179.994	Co1—O1—H12	121.0
N1—Co1—O1	87.29 (6)	H11—O1—H12	105.0
N2—Co1—O1	91.15 (6)	F1—B1—F2	109.0 (6)
Co1—N1—C1	173.92 (16)	F1—B1—F3	105.3 (7)
Co1—N2—C6^ii^	166.34 (18)	F2—B1—F3	109.1 (7)
N1—C1—C2	179.5 (2)	F1—B1—F4	108.3 (6)
C1—C2—C3	113.2 (2)	F2—B1—F4	115.9 (6)
C1—C2—H21	108.5	F3—B1—F4	108.7 (6)
C3—C2—H21	109.3	F1—B1—F21	105.8 (6)
C1—C2—H22	108.7	F1—B1—F31	115.0 (6)
C3—C2—H22	109.9	F21—B1—F31	109.8 (7)
H21—C2—H22	107.0	F1—B1—F41	108.4 (6)
C2—C3—C4	125.87 (19)	F21—B1—F41	106.8 (6)
C2—C3—H31	115.5	F31—B1—F41	110.7 (8)

Symmetry codes: (i) −*x*, −*y*+2, −*z*+1; (ii) −*x*+1, −*y*+1, −*z*+1.

### Hydrogen-bond geometry (Å, º)

**Table:**

*D*—H···*A*	*D*—H	H···*A*	*D*···*A*	*D*—H···*A*
C2—H22···O1^iii^	0.97	2.52	3.348 (3)	143 (1)
O1—H12···F1^iv^	0.83	1.89	2.72 (2)	175 (1)
O1—H11···F2^iii^	0.82	1.87	2.669 (13)	163 (1)

Symmetry codes: (iii) −*x*+1, −*y*+2, −*z*+1; (iv) *x*−1, *y*+1, *z*.
