# Supplementary figures and images for: Crystal structure of catena-poly[[[di­aqua­cobalt(II)]-bis­(μ-hex-3-enedi­nitrile-κ2 N:N′)] bis­(tetra­fluorido­borate)]
Source: Acta Crystallogr E Crystallogr Commun. 2015 May 23;71(Pt 6):m135–6. doi: 10.1107/S2056989015009548 (PMC4459353; doi:10.1107/S2056989015009548)

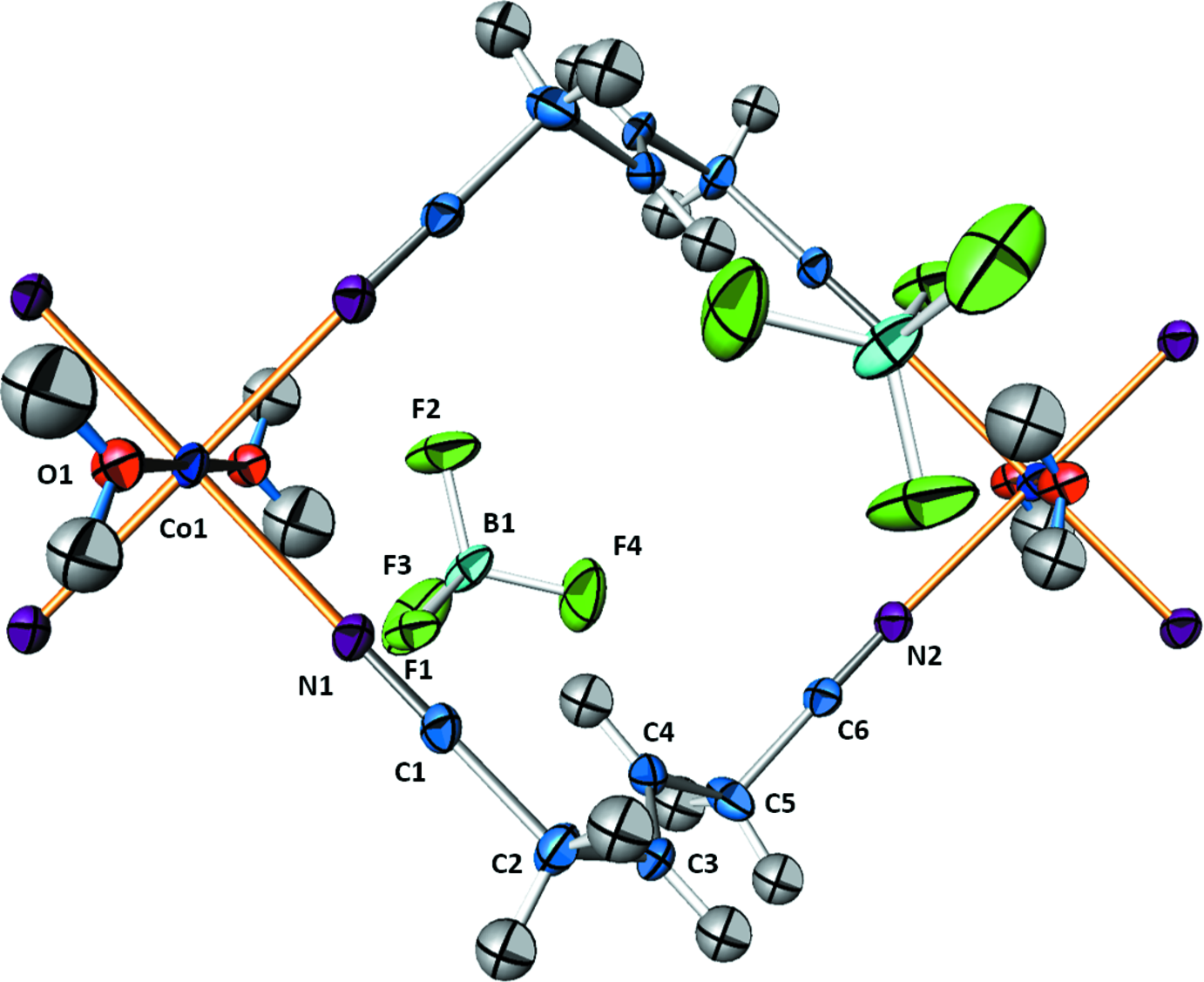

Supplement: Supplementary file 3 [file e-71-0m135-fig1.tif]

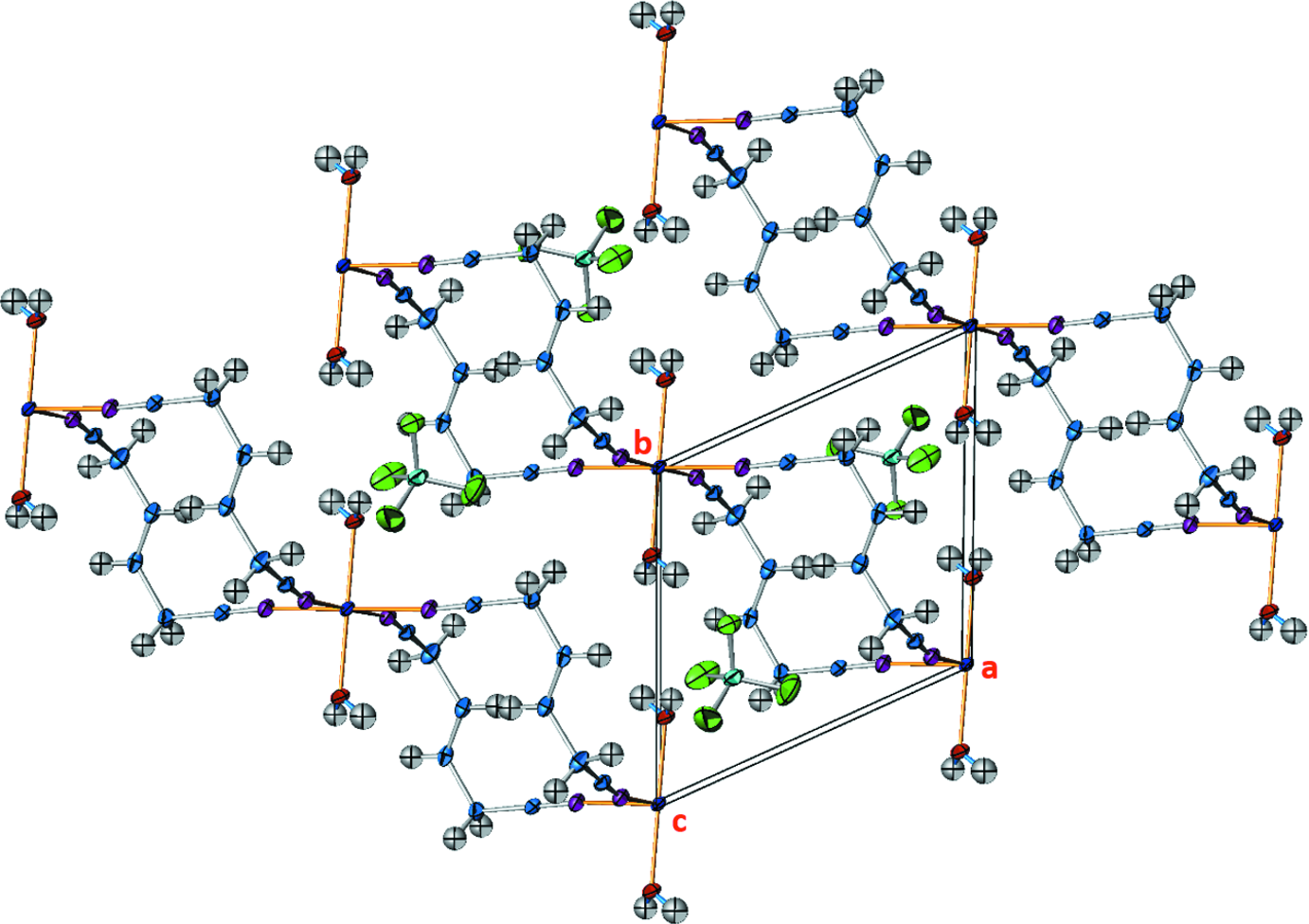

Supplement: Supplementary file 4 [file e-71-0m135-fig2.tif]
